# Ribosomal Protein S27-Like in Colorectal Cancer: A Candidate for Predicting Prognoses

**DOI:** 10.1371/journal.pone.0067043

**Published:** 2013-06-24

**Authors:** Chi-Jung Huang, Shung-Haur Yang, Chia-Long Lee, Yu-Che Cheng, Szu-Yun Tai, Chih-Cheng Chien

**Affiliations:** 1 Department of Medical Research, Cathay General Hospital, Taipei, Taiwan; 2 School of Medicine, Fu Jen Catholic University, New Taipei, Taiwan; 3 Department of Biochemistry, National Defense Medical Center, Taipei, Taiwan; 4 Department of Surgery, Taipei-Veterans General Hospital, Taipei, Taiwan; 5 School of Medicine, National Yang Ming University, Taipei, Taiwan; 6 Department of Internal Medicine, Hsinchu Cathay General Hospital, Hsinchu, Taiwan; 7 Department of Internal Medicine, School of Medicine, College of Medicine, Taipei Medical University, Taipei, Taiwan; 8 Department of Anesthesiology, Sijhih Cathay General Hospital, New Taipei, Taiwan; Health Canada and University of Ottawa, Canada

## Abstract

**Background:**

The development and progression of colorectal cancer (CRC) involve a complex process of multiple genetic changes. Tumor suppressor p53 is capable of determining the fate of CRC cells. However, the role of a p53-inducible modulator, ribosomal protein S27-like (RPS27L), in CRC is unknown.

**Methods:**

Here, the differential expression of RPS27L was examined in the feces and colonic tissues of CRC patients, to explore its possible correlation with patient survival and to investigate the cellular mechanisms underlying their clinical outcomes. Eighty intermediate-stage CRC patients (42 at stage II and 38 at stage III) were divided into two groups according to their fecal RPS27L mRNA levels. The survival probabilities of the groups were estimated using the Kaplan–Meier method. The RPS27L protein in the colonic tissues of stage III patients with different prognoses was further examined immunohistochemically. RPS27L expression in LoVo cells was manipulated to examine the possible cellular responses in vitro.

**Results:**

Elevated RPS27L expression, in either feces or tissues, was related to a better prognosis. In vitro, RPS27L-expressing LoVo cells ceased DNA synthesis and apoptotic activity while the expression of their DNA repair molecules was upregulated.

**Conclusions:**

Elevated RPS27L may improve the prognoses of certain CRC patients by enhancing the DNA repair capacity of their colonic cells, and can be determined in feces. By integrating clinical, molecular, and cellular data, our study demonstrates that fecal RPS27L may be a useful index for predicting prognoses and guiding personalized therapeutic strategies, especially in patients with intermediate-stage CRC.

## Introduction

The development and progression of colorectal cancer (CRC), one of the most common fatal cancers, involve a complex process of multiple genetic changes [1], [2]. Surgery is the optimal treatment for CRC patients at stages II and III, but adjuvant chemotherapy has improved the prognosis for some of these intermediate-stage patients [3]. However, despite treatment, up to 25% of patients at stage II and 30–40% at stage III develop a distant metastasis or local relapse [4]. Molecular markers of CRC can be used to improve the decisions made regarding adjuvant chemotherapy in these patients [5], but remain controversial [3].

When cells encounter stresses, tumor suppressor p53 is capable of determining their fate by facilitating the repair and survival of damaged cells or by eliminating severely damaged cells [6]. CRC tumorigenesis has long been related to functional loss of p53 and the consequent changes in expression of p53 responsive genes [7]. The most studied of these p53-responsive effects is the repair of damaged DNA, which is believed to be a major contributor to tumor progression [8]. The detection of alterations in expression of p53-responsive genes has been suggested to allow identification of patients at high risk of recurrence and those who should be considered for adjuvant chemotherapy [9].

A group of ribosomal proteins with clinical significance to many human cancers has been identified, and the genes encoding most of these are responsive to p53 [10], [11]. In fact, in addition to their role in assembling with rRNA to construct ribosomes for new protein synthesis, ribosomal proteins are known to have many extraribosomal roles [12]–[14]. One of the ribosomal proteins, ribosomal protein S27-like (RPS27L), was reported to be downregulated in feces and tumor tissues of some late-stage CRC patients [15], [16]. This ribosomal protein and its homologous protein, RPS27, have been considered to have extended roles in cell growth regulation and DNA repair [17], [18]. In addition, RPS27L has been identified as a p53-inducible modulator of cell fate [19], [20]. Therefore, we investigated the clinical meaning and cellular effects of RPS27L expression and the possible mechanisms underlying its involvement in the clinical outcomes of CRC.

We used quantitative real-time RT–PCR (qRT–PCR) to quantify the heterogeneity of fecal RPS27L levels in intermediate-stage CRC patients, and the differential expression of RPS27L was correlated with their clinical outcomes. RPS27L expression in LoVo cells with wild-type p53 was manipulated to examine the possible cellular responses in vitro by analyzing the changes in the cell phases, their apoptotic functions, and DNA repair.

## Materials and Methods

### Fecal and Tissue Specimens

Solid fecal samples from 80 sporadic CRC patients (42 at stage II and 38 at stage III), obtained at the Cathay General Hospital or Taipei Veterans General Hospital, were collected before surgery or any chemotherapy. Fecal total RNA was prepared according to our previously reported protocol, using an RNA extraction kit (Bioman Scientific, Taipei, Taiwan) with some modifications [21] (Methods S1). The cell phases in the CRC tissue samples (n = 68) and the patient survival data (n = 71) were acquired from the patients whose fecal samples were analyzed. Another six stage III CRC tissues were collected for immunohistochemical staining to determine the expression of p53 and RPS27L proteins. The study was reviewed and approved by the Institutional Review Board of Cathay General Hospital. The ethics committees, the Institutional Review Board of Cathay General Hospital, approved this consent procedure. All participants provided their written informed consent to participate in this study. Follow-up data were obtained prospectively, and the mean follow-up time was 39.0 months (SD, 3.1; median, 28.3).

### Lentivirus-based RNA Interference (RNAi) and Overexpression of RPS27L

The lentiviral particles generated to silence RPS27L (NM_015920) (pLK0.1-RPS27L, TRCN0000117608: GCCTAGTACAAAGTCCAAATT), to silence RPS27 (NM_001030) (pLK0.1-RPS27, TRCN0000117596: GAAGAGGAAACACAAGAAG), or to overexpress RPS27L (pLKO AS3w.puro, containing RPS27L cDNA) were obtained from the National RNAi Core Facility located at the Institute of Molecular Biology/Genomic Research Center, Academia Sinica, Taiwan. pLK0.1-Luc (TRCN0000072246) was used as the control. Various colonic cell lines were infected with each lentivirus using the protocol of the RNAi Core Facility. Briefly, 8.5×10^5^ colonic cells were grown in a 10 cm plate for 24 hours, and then infected with lentivirus at a multiplicity of infection of 3. Stable infected cells were selected and maintained in medium containing 2 µg/mL puromycin (Invitrogen, Carlsbad, CA). Changes in the expression of the target genes were determined with qRT–PCR or immunoblotting (Methods s1).

### Apoptosis Assay

At 24 hours before the VP16 (also known as etoposide or VP-16-213; a topoisomerase II inhibitor) treatment, the cells were seeded into 16-well chamber slides at a density of 5×10^3^ cells per well. Either RPS27L-expressing (shLuc) or RPS27L-lacking (shRPS27L) LoVo cells were treated with 10 µM VP16 for 48 hours. To identify apoptosis-positive cells, the cells were stained with an annexin-V/PI double staining assay, with the FITC Annexin V Apoptosis Detection Kit I (BD Biosciences), according to the manufacturer’s protocol, with a minor modification. Briefly, the cells were washed with wash buffer (20 mM Tris [pH 7.4], 150 mM NaCl, 1 mM CaCl_2_) after incubation for 15 minutes in binding buffer containing 5 µL of annexin V–FITC and 5 µL of PI at room temperature in the dark. Additional evidence for the occurrence of apoptosis was obtained using the mitochondrial membrane potential (ΔΨ) and cleaved poly(ADP-ribose) polymerase (PARP). The variations in ΔΨ were revealed via microscopic fluorescence using a MitoLight Apoptosis Detection Kit (Millipore, Billerica, MA) containing 5,5′,6,6′-tetrachloro-1,1′,3,3′-tetraethylbenzimidazolylcarbocyanine iodide (JC-1), according to the manufacturer’s instructions, with minor modifications. First, 6×10^4^ colonic cells per well were incubated and treated with VP16, as described above. After incubation for 24 hours with VP16, each sample was treated with diluted MitoLight reagent (0.2 µL of MitoLight and 180 µL of deionized water) and 20 µL of 10× incubation buffer for 15 minutes at 37°C in a humidified atmosphere containing 5% CO_2_. The highly negative membrane potential in the mitochondria produces orange-red fluorescence from JC-1, and the loss of the mitochondrial transmembrane potential results in green fluorescence, with the loss of the red fluorescence. The levels of cleaved PARP protein were determined on immunoblots (Methods s1).

### γ-H2AX Foci Formation Assay

After 5×10^4^ LoVo cells per well had been incubated for 24 hours, DNA double-stranded breaks (DSBs) were induced in these cells with 10 µM VP16. The cells’ ability to repair the DSBs was determined by staining them with anti-γ-H2AX antibody using the OxiSelect DNA Double Strand Break Staining Kit (Cell Biolabs, San Diego, CA), according to the manufacturer’s instructions, with some minor changes. Briefly, the cells were allowed to recover by removing the DSB inducer for the indicated periods. The DSBs revealed (as γ-H2AX foci) appeared as green fluorescence, and the nuclei were visualized by counterstaining them with DAPI (4′-6-Diamidino-2-phenylindole). The green fluorescence indicating γ-H2AX foci was visualized with Adobe Photoshop CS4 Extended (ver. 11.0; Adobe Systems, San Jose, CA) in 100–200 randomly chosen cells. A threshold was determined by measuring similar levels of green color in cells that had recovered over different periods (0 hours, 2 hours, or 4 hours). The results are presented as the percentages of cells emitting fluorescence above the threshold level.

### Statistical Analysis

Survival curves and survival probabilities were estimated using the Kaplan–Meier method and compared using the log-rank test. Variables with *P*-values ≤ 0.1 in the univariate analyses were included in the multivariate model of the Cox proportional hazards analysis, using an enter method for survival analysis. Differences in gene expression and in the numbers of the γ-H2AX foci were compared using Student’s t test. Statistical analyses were performed with SPSS 13.0 software (SPSS, Somers, NY). The MedCalc statistical software package (MedCalc, Mariakerke, Belgium) was used to generate the receiver operating characteristic (ROC) curves. The data shown here are representative of at least three experiments with similar results, and *P*-values <0.05 are considered significant.

## Results

### Clinical Significance of RPS27L in CRC

**Figure 1:**
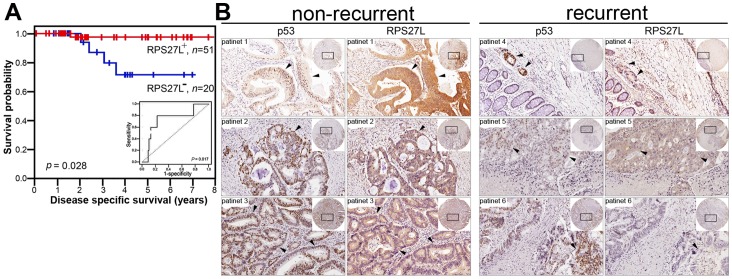
Expression levels of RPS27L were analyzed in feces and CRC tissues of CRC patients. (A) Clinical meaning of RPS27L mRNA in feces. Levels of RPS27L mRNA was relatively quantified by qRT-PCR. The points on the receiver operating characteristic curve (black inset) represented the sensitivity and (1-specificity) for DSS. Kaplan–Meier estimated of DSS dependent on RPS27L mRNA levels (RPS27L–, <0.0014; RPS27L+, (0.0014) in feces of CRC patients. (B) Protein levels of p53 and RPS27L in CRC tissues. Each sample was stained with anti-p53 and anti-RPS27L antibodies from two adjacent sections. Non-recurrent patients: patients 1 to 3, survival >8 y; Recurrent patients: patients 4 to 6, survival <4 y. All slides were counterstained with hematoxylin. The same field of the inset at low magnification (40×) was shown at higher magnification (200×). Arrows, the corresponding positions for each patient.

The level of fecal RPS27L correlated positively with the percentage of CRC cells of intermediate-stage CRC patients in S phase (r_s_ = 0.259; *P* = 0.033, Pearson correlation test). We performed a ROC curve analysis and stratified the patients into two groups using the fecal RPS27L level of 0.0014, for which a sensitivity of 0.80 (95% confidence interval [CI], 0.28–0.99) and a specificity of 0.77 (95% CI, 0.66–0.86) were achieved, to predict the prognosis of patients. The area under the ROC curve for fecal RPS27L was 0.733 (*P* = 0.017), with a 95% CI of 0.623–0.826 (Figure 1A). The survival rates were compared between the RPS27L^+^ (n = 51; ≥ 0.0014) and RPS27L^−^ groups (n = 20; <0.0014); the five-year disease-specific survival (DSS) rate was higher in the RPS27L^+^ group (97.1% ±2.8%) than in the RPS27L^−^ group (69.6% ±12.9%; P = 0.028, log-rank test). No relationship was evident between the RPS27L^+^ group and other clinicopathological parameters (Table 1). However, univariate and multivariate Cox proportional hazards analyses showed that the fecal RPS27L level was an independent prognostic factor (hazards ratio, 0.086; 95% CI, 0.009–0.852; *P* = 0.036) for DSS of intermediate-stage CRC patients (Table 2). In addition, three nonrecurrent patients (stage III) with favorable prognoses (survival >8 years) displayed intense immunohistochemical staining for p53 and RPS27L in tissue sections (Figure 1B, patients 1–3). Three other stage III patients, who suffered recurrence within one year after surgery and died after the first recurrence (survival <4 years), were also positive for p53 protein in their tumor cells, but the expression of RPS27L protein was weak at the corresponding positions (Figure 1B, patients 4–6).

**Table 1:** Clinical characteristics of CRC patients according to levels of fecal RPS27L.

Variable	n	≧ 0.0014 (%)	*P*
**Age (years)**	72		0.148
** < 66.7**		22/35 (62.9)	
** ≧ 66.7**		29/37 (78.4)	
**Gender**	74		0.253
** Male**		33/49 (67.3)	
** Female**		20/25 (80.0)	
**Tumor site**	74		0.344
** Right colon**		15/22 (68.2)	
** Left colon**		22/27 (81.5)	
** Rectum**		16/25 (64.0)	
**Dukes’ stage**	80		0.217
** II**		31/40 (77.5)	
** III**		26/40 (65.0)	
**Tumor size (cm)**	72		0.463
** < 4.8**		33/43 (76.7)	
** ≧ 4.8**		20/29 (69.0)	
**Tumor growth**	74		0.427
** Expansive**		7/8 (87.5)	
** Infiltrative**		46/66 (69.7)	
**Histological differentiation**	72		1
** Well/moderate**		49/67 (73.1)	
** Poor/undifferentiated**		4/5 (80.0)	
**Mucinous component**	73		1
** ≦ 50%**		50/70 (71.4)	
** > 50%**		2/3 (66.7)	
**CEA (ng/ml)**	70		0.931
** ≦ 5**		37/50 (74.0)	
** > 5**		15/20 (75.0)	
**CA19-9 (U/ml)**	71		1
** < 37**		42/57 (73.7)	
** ≧ 37**		11/14 (78.6)	

**Table 2:** Cox proportional hazards analysis of disease-specific survival.

	Hazard ratio (95% CI)	*P*
**Fecal RPS27L (0.0014)**	0.086 (0.009–0.852)	0.036
**Gender (male, female)**	0.141 (0.017–1.141)	0.066
**Stage (II, III)**	7.260 (0.706–74.645)	0.095

### Translocation of RPS27L in Colorectal Cancer Cells

**Figure 2:**
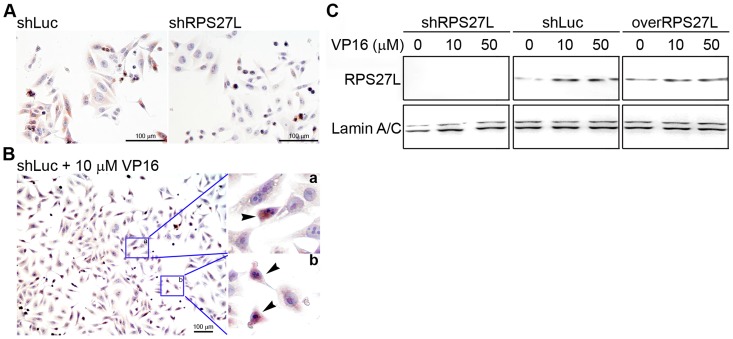
Immunodetection of RPS27L in LoVo cells. (A) Cellular localization of RPS27L. RPS27L was immuno-stained in various LoVo cells. (B) Nuclear induction of RPS27L using 10 (M VP16. Arrows in insets (a and b) indicated condense nuclear RPS27L-stains. (C) Immuno-detection of RPS27L in various nuclear extracts. The nuclear extract was prepared from synchronized LoVo cells with VP16 as indicated. shLuc, RPS27L-expressing cells; shRPS27L, RPS27L-lacking cells, overRPS27L, RPS27L-overexpressing cells. Anti-RPS27L antibody, 1∶2000.

To investigate the cellular function of RPS27L, we infected LoVo cells, a wild-type p53-expressing cell line from a stage at stage III CRC, using a lentivirus-mediated short hairpin RNA (shRNA) to efficiently knock down RPS27L expression (shRPS27L) or a control shRNA (shLuc) (Figure S1). Immunocytochemical staining for endogenous RPS27L was positive in the cytoplasm of the shLuc-infected LoVo cells but was negative in the cells infected with shRPS27L (Figure 2A). The cytoplasmic RPS27L was translocated into the nuclei of 10 µM VP16-treated LoVo cells, and the intense staining for RPS27L protein in the nuclei is shown in Figure 2B. This VP16-induced change was also observed during the immunodetection of RPS27L in the original LoVo cells (Figure 2C).

### Cellular Effects of Down-regulated RPS27L in Colorectal Cancer Cells

**Figure 3:**
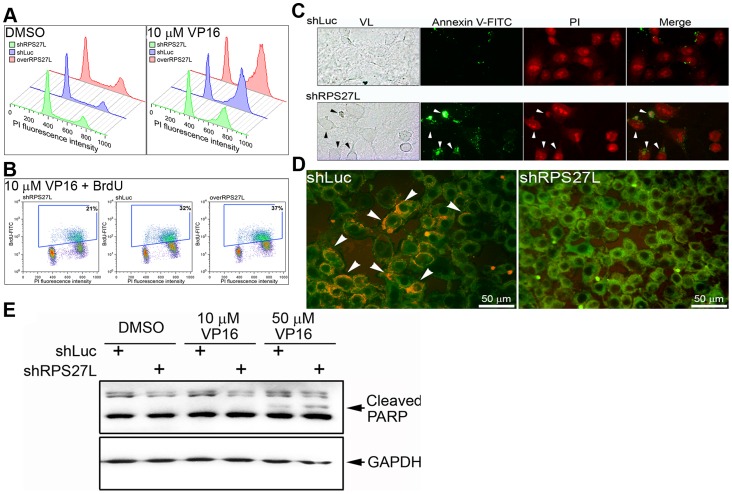
Cellular effects of differentially expressed RPS27L in LoVo cells. (A) Cell cycle regulation. Cell phases were determined following an indicated VP16 treatment. (B) Cells with newly synthesized DNA under VP16 treatment. The percentage of BrdU-positive cells (blue spots) was determined following an indicated VP16 treatment. (C) Annexin-V/PI double-staining assay. Arrows indicated annexin V-FITC and PI positive cells. VL, visible light; PI, propidium iodide. Green spot, annexin V-FITC; red spot, PI. (D) Cells with JC-1 stain. Arrows indicated the red granular pattern of JC-1 aggregation. (E) Immuno-detection of PARP cleavage. Cleaved PARP, 89 kDa; GAPDH, 36 kDa. shLuc, RPS27L-expressing cells; shRPS27L, RPS27L-lacking cells; overRPS27L, RPS27L-overexpressing cells.

We next analyzed the cellular effects of RPS27L by overexpressing RPS27L in LoVo cells (overRPS27L) (Figure S1). Similar cell-cycle regulation was observed in the VP16-free cells (independent of RPS27L expression) as reported in previous studies (Figure 3A) [22]. When the DNA of the synchronized cells was damaged by VP16 in complete medium, the numbers of cells in the S and G_2_/M phases increased according to the expression of RPS27L (shLuc or overRPS27L). However, another similar RP, *RPS27*, did not alter the size of the S-phase population, regardless of the level of *RPS27* expression following VP16 treatment (Figure S2). Simultaneous measurements of the DNA content and the amount of BrdU-labeled DNA indicated that there were fewer cells with active DNA synthesis among the RPS27L-lacking cells (21% in shRPS27L) following treatment with 10 µM VP16 (Figure 3B). In contrast, both the RPS27L-expressing and RPS27L-overexpressing cells had a higher proportion of cells with active DNA synthesis (32% in shLuc and 37% in overRPS27L) under identical conditions (Figure 3B).

The apoptotic activity of RPS27L in LoVo cells was assessed. Briefly, the control cells (shLuc) were negative for annexin V–FITC. The RPS27L-lacking cells (shRPS27L) contained more annexin-V-positive cells and displayed a late apoptotic (or necrotic) stage when doubly positively stained (annexin V and PI) (Figure 3C). Moreover, living cells expressing RPS27L were detected using the red granular pattern of JC-1 aggregation in cells treated with 10 µM VP16 (Figure 3D) and increased levels of cleaved PARP were observed with increasing concentrations of VP16 (Figure 3E). In the RPS27L-lacking cells, this red granular pattern did not exist and the highest level of cleaved PARP was observed (Figures 3D and 3E).

### DNA Repair Function of RPS27L in Colorectal Cancer Cells

**Figure 4:**
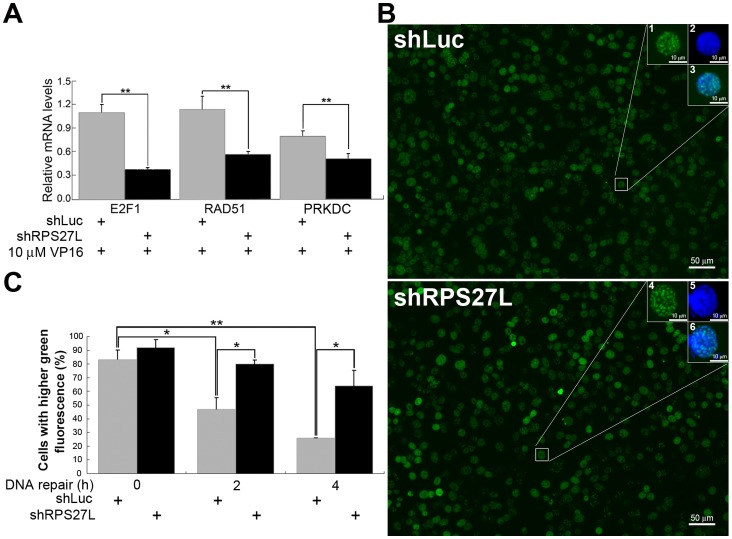
Effects of RPS27L in DNA repair of VP16-induced double-strand breaks in LoVo cells. (A) Relative expressions of DNA repair genes. The mRNA levels of *E2F1, RAD51*, and *PRKDC* were quantified using qRT-PCR following cells with indicated VP16 treatment. The relative expression of each gene was calculated by comparing that of shLuc cells without VP16 treatment. (B) Representative photos of cells with γ-H2AX foci. Cells were allowed to recover by removing the DSBs inducer for 4 h (100×). A threshold was determined by measuring similar levels of green fluorescence (γ-H2AX foci, uncovered DSBs) between shLuc and shRPS27L cells. Insets (200×): 1 and 4, γ-H2AX foci (also indicated the threshold); 2 and 5, DAPI; 3, merge of 1 and 2; 6, merge of 4 and 5. (C) Statistical difference of γ-H2AX foci in cells. Cells were treated with 10 µM of VP16 for 1 h and recovered for indicated time. Percentage of 100–200 randomly chosen cells whose green fluorescence were higher than threshold were indicated. shLuc, RPS27L-expressing cells; shRPS27L, RPS27L-lacking cells. Data are mean ± SD. *, *P*<0.05; **, *P*<0.01. Two-to-three independent experiments were performed.

We further quantified the mRNA expression of E2F transcription factor 1 (E2F1), RAD51, and the protein kinase, DNA-activated, catalytic polypeptide (PRKDC) to identify the role of RPS27L in VP16-induced DSBs. In RPS27L-lacking cells, treatment with 10 µM VP16 induced a 63.6% reduction (1.1- to 0.4-fold) in E2F1 expression and a 54.5% reduction (1.1- to 0.6-fold) in RAD51 expression (Figure 4A). Similarly reduced expression of PRKDC (0.8- to 0.5-fold) was also observed under the same treatment conditions. The percentage of cells with intense phosphorylated histone H2AX (γ-H2AX) foci decreased gradually as the repair time increased from 0 to 4 hours, regardless of whether the LoVo cells expressed RPS27L (Figure 4B). However, a delay in the kinetics of the decreasing intensity of γ-H2AX foci was observed in cells lacking RPS27L expression. Up to 61% of RPS27L-lacking cells contained a high density of γ-H2AX foci after repair for 4 hours, whereas only 17% of RPS27L-expressing cells were above the density threshold for γ-H2AX foci at that time (Figure 4C).

## Discussion

Molecular markers may constitute a better basis for selecting therapy than clinical and histopathological criteria [23]. Carcinoembryonic antigen (CEA) and cancer antigen 19–9 (CA19–9) are the most widely used markers of CRC [24]. However, high levels of either serum CEA or CA19–9 are considered to indicate an unfavorable prognosis in CRC patients with intermediate-stage disease [25], [26].

In the present study, we focused on CRC patients in the intermediate stages of their disease because adjuvant therapy is important for these patients. We previously reported that the expression of numerous RPs, including RPS27L, differed significantly in the feces of CRC patients; but no significant expression difference was found for RPS27, a member of the same family as RPS27L [15]. We have now shown that intermediate-stage patients with increased fecal RPS27L expression had a higher DSS rate and a much lower hazards ratio.

Currently, we monitor functional p53 by determining the fecal RPS27L status of patients with intermediate-stage CRC. The clinical significance of RPS27L was not only demonstrated in fecal samples, but also in CRC tissue samples. In intermediate-stage CRC, intense RPS27L staining, with an increase in p53 protein, in the CRC tissue was found in patients displaying longer survival. However, patients whose p53 protein failed to activate the expression of RPS27L had poor prognoses, with recurrence and shorter survival. This implies that intermediate-stage CRC patients with higher RPS27L levels, whether in the feces or tissues, have a more favorable prognosis, and consistent expression of p53 and RPS27L was observed in these patients with better prognoses.

This is the first report showing that RPS27L is a potential prognostic marker for CRC. In our clinical samples, the level of fecal RPS27L correlated positively with the percentage of CRC cells in S phase. These clinical data are consistent with our results from synchronized LoVo cells that expressed RPS27L. RPS27L and its homologous protein, RPS27, can interact with the p53–MDM2 axis in various cell types [20]. In the present study, we used RNA interference (shRPS27L) to knock down RPS27L expression in LoVo cells. We found that no difference in RPS27 expression was detected from the cells with or without RPS27L silencing (our unpublished data) and no consistent cellular effect was observed in RPS27L- and RPS27-lacking LoVo cells. Further, shRPS27L does not affect the expression of any other genes, even those (klotho, NM_004795; archaelysin family metallopeptidase 1, NM_133463) with mRNA sequences that have partial matches to shRPS27L (Figure S3). In fact, klotho and archaelysin family metallopeptidase 1 are naturally expressed at low levels in our target CRC cell line, LoVo (data not shown) [27], [28]. Overall, our results confirm that the effects of shRPS27L are specific. In addition, the protein sequence of RPS27L was checked against the human genome in the UCSC genome server [29]. Twenty-nine protein sequences with various degrees of identity (61.6%–98.8%) to RPS27L were identified, but none of these were knocked down by shRPS27L, because of its specific sequence. This once again emphasizes that no protein other than RPS27L can be knocked down by shRPS27L.

Following the confirmation of the specificity of action of shRPS27L, we speculated that p53-responsive RPS27L causes the arrest of the cells in S phase when the cellular DNA is damaged. After DNA damage and p53 were induced by the addition of VP16, BrdU-positive cells in S phase accumulated among the RPS27L-expressing LoVo cells. These findings suggest that RPS27L causes the cells to arrest in S phase following active DNA synthesis, and does not allow these cells to enter M phase. The observation of annexin-V-positive and JC-1-aggregated cells strongly suggests that the DNA-damaging agent, VP16, induces an antiapoptotic effect in cells expressing both wild-type p53 and RPS27L. This antiapoptotic effect was also reflected in the higher levels of cleaved PARP in the RPS27L-lacking cells. The activation of PARP is also essential for the regulation of many cellular processes, including DNA repair and cell death [30].

However, the active DNA synthesis and antiapoptotic effect observed, even when the cellular DNA was damaged, seem incompatible with our clinical data, indicating that patients with higher levels of RPS27L expression have a more favorable prognosis. We hypothesize that nonapoptotic cells with damaged DNA must access a suitable method for DNA DSB repair, and that targeting DNA repair proteins should be widely used in cancer treatment [31]. This can be illustrated by the quantification of two DNA DSB repair genes, RAD51 and PRKDC [32], and one gene that encodes a component of repair complexes, E2F1 [33]. Their reduced expression coincided with RPS27L knockdown after treatment with low-dose VP16. This is consistent with the results that E2F1 plays a functional role in suppressing apoptosis and promoting DNA repair in vivo after DNA damage [34]. Consequently, RPS27L is required to suppress apoptosis and for DNA DSB repair in colonic cells expressing wild-type p53. Moreover, VP16-induced DSBs can induce the accumulation of RPS27L protein in the nuclei of LoVo cells. This suggests that DNA DSB damage plays a critical role in inducing the movement of RPS27L protein from the cytoplasm to the nucleus. Xiong et al. also reported similar results in a study of human lung adenocarcinoma epithelial cells [20]. We speculate that the translocation of RPS27L protein might be crucial in the regulation of some DNA repair genes. However, further studies are required to clarify the relationship between RPS27L expression and DNA DSB repair genes.

The crucial regulation of RPS27L in DNA DSB repair can also be assessed by measuring the kinetics of the changes in the numbers of γ-H2AX foci [35]. In combination with the results for BrdU incorporation, we suggest that RPS27L participates in DNA DSB repair through a mechanism of homologous recombination, because most BrdU-positive cells were restricted to late S phase [36]. This partly corresponds to the findings of Miquel et al., who showed that a high proportion of CRC cells are unable to repair DNA because of changes in one or more components of the two main DNA-repairing mechanisms, including the homologous recombination pathway [37].

### Conclusions

Elevated p53-responsive RPS27L accumulation in the nucleus may improve the prognoses of certain CRC patients, possibly through its effect on DNA repair in colonic cells. RPS27L accumulation can be observed in the feces. By integrating clinical, molecular, and cellular data, our study has demonstrated that fecal RPS27L may be an index of prognosis in CRC patients and may guide personalized therapeutic strategies, especially in patients with intermediate-stage CRC.

## Supporting Information

Figure S1 **Efficient changes of RPS27L expression by conducting lentivirus-mediated experiments in LoVo cells.** Stable RPS27L-expressing (shLuc), RPS27L-lacking (shRPS27L), and RPS27L-overexpressing (overRPS27L) LoVo cells were acquired by various Lentivirus infections. RPS27L, 9 kDa; GAPDH, 36 kDa.
(TIF)

Figure S2 **Effect of RPS27 expression on flow cytometry.** LoVo cells, which either silenced RPS27 (shRPS27) or infected the control virus (shLuc) were treated with DMSO or 10 µM of VP16. Sorting analyses of propidium iodide (PI)-stained cells by flow cytometry. Approximately 104 cells in different phases of the cell cycle were determined using FlowJo 8.7 software.
(TIF)

Figure S3 **mRNA sequences with partial matches to shRPS27L.** shRPS27L was the RNA interference to knock down RPS27L expression. Each dot meant the identical nucleotide with the sequence of shRPS27L. Ranges of matched sequences in each cDNA were indicated. Ribosomal protein S27-like (RPS27L): NM_015920; klotho: NM_004795; archaelysin family metallopeptidase 1 (AMZ1): NM_133463.
(TIF)

Methods S1 (DOC)
