# Supplementary figures and images for: Ribosomal Protein S27-Like in Colorectal Cancer: A Candidate for Predicting Prognoses
Source: PLoS One. 2013 Jun 24;8(6):e67043. doi: 10.1371/journal.pone.0067043 (PMC3691124; doi:10.1371/journal.pone.0067043)

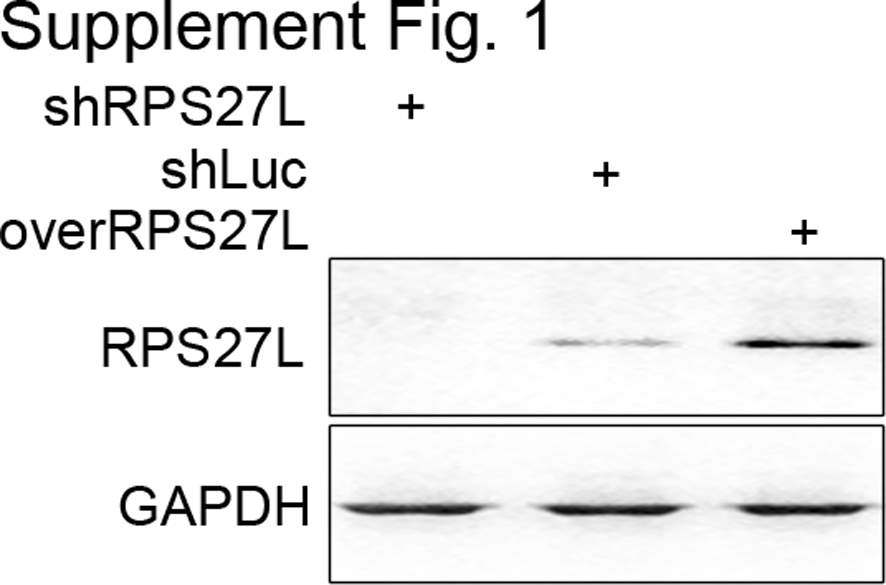

Supplement: Figure S1 — Efficient changes of RPS27L expression by conducting lentivirus-mediated experiments in LoVo cells. Stable RPS27L-expressing (shLuc), RPS27L-lacking (shRPS27L), and RPS27L-overexpressing (overRPS27L) LoVo cells were acquired by various Lentivirus infections. RPS27L, 9 kDa; GAPDH, 36 kDa. (TIF) [file pone.0067043.s001.tif]

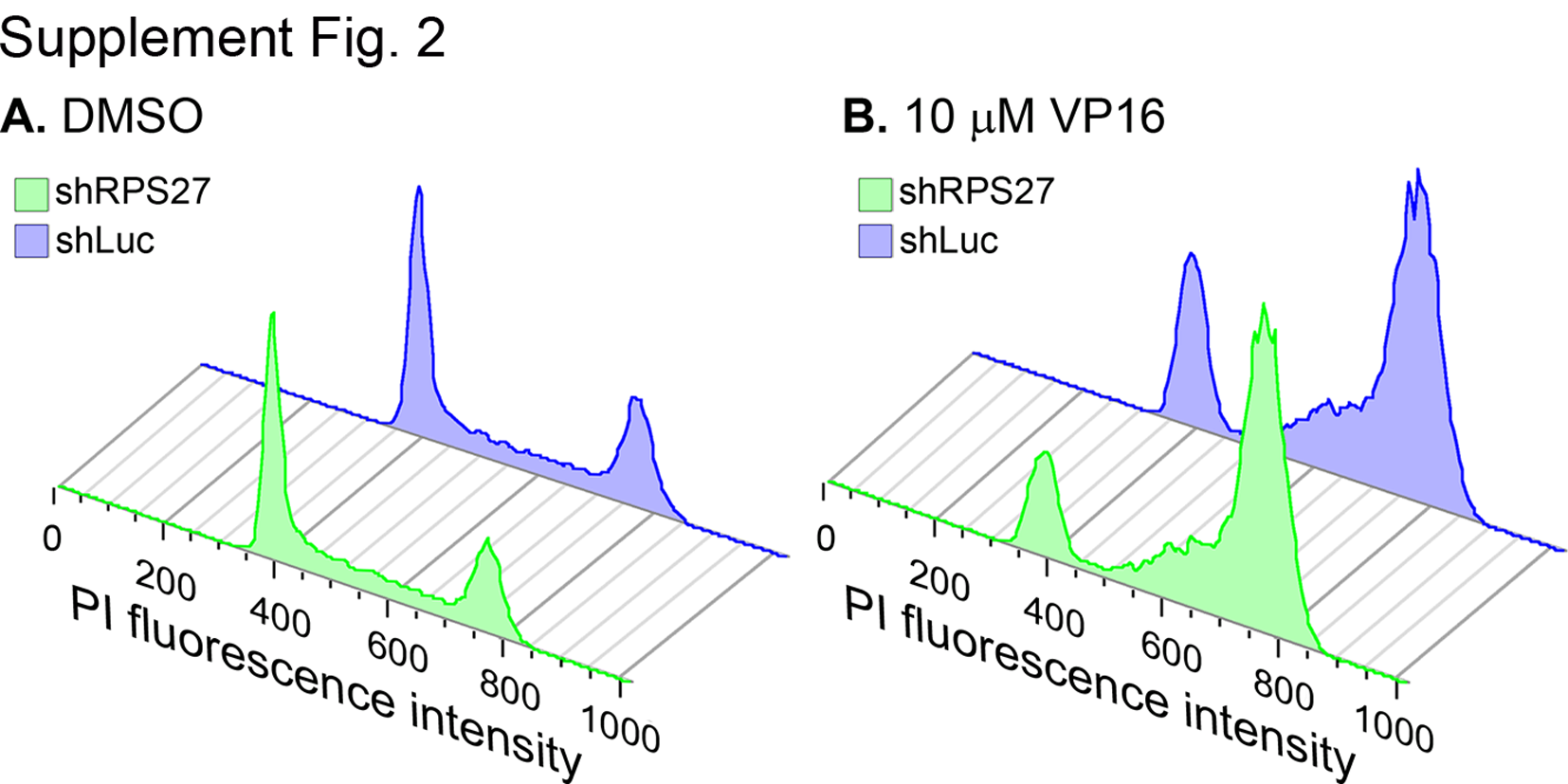

Supplement: Figure S2 — Effect of RPS27 expression on flow cytometry. LoVo cells, which either silenced RPS27 (shRPS27) or infected the control virus (shLuc) were treated with DMSO or 10 µM of VP16. Sorting analyses of propidium iodide (PI)-stained cells by flow cytometry. Approximately 104 cells in different phases of the cell cycle were determined using FlowJo 8.7 software. (TIF) [file pone.0067043.s002.tif]

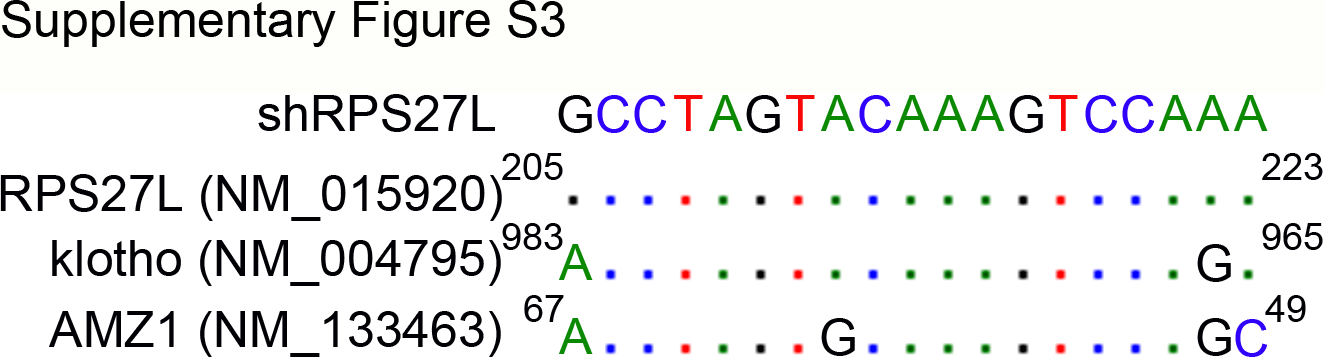

Supplement: Figure S3 — mRNA sequences with partial matches to shRPS27L. shRPS27L was the RNA interference to knock down RPS27L expression. Each dot meant the identical nucleotide with the sequence of shRPS27L. Ranges of matched sequences in each cDNA were indicated. Ribosomal protein S27-like (RPS27L): NM_015920; klotho: NM_004795; archaelysin family metallopeptidase 1 (AMZ1): NM_133463. (TIF) [file pone.0067043.s003.tif]
