# Supplementary material for: Ribosomal Protein S27-Like in Colorectal Cancer: A Candidate for Predicting Prognoses
Source: PLoS One. 2013 Jun 24;8(6):e67043. doi: 10.1371/journal.pone.0067043 (PMC3691124; doi:10.1371/journal.pone.0067043)
Supplement: Methods S1 — (DOC) [file pone.0067043.s004.doc]

**Supplementary Methods**

## Fecal Total RNA Preparation

## Each fecal sample (approximately 0.5 g) was preserved in 1 mL of guanidinium thiocyanate buffer (10 mM Tris [pH 7.4], 200 mM NaCl, 1 mM EDTA, [pH 8.0], 4 M guanidinium thiocyanate, 1% β-mercaptoethanol). The fecal mud was then homogenized on a rocking platform for 30 minutes at room temperature, and the debris was removed by two centrifugations (8,000 × g for 15 minutes and 20,000 × g for 20 minutes) at 4 °C. An aliquot (800 μL) of this supernatant was then mixed with 680 μL of RB buffer and 500 μL of absolute alcohol. The fecal total RNA was allowed to bind to the RNA column. Before elution, the column was washed twice with 500 μL of W1 buffer and three times with 500 μL of wash buffer. The eluted fecal total RNA was then quantified using a NanoDrop ND 1000 spectrophotometer (Thermo Fisher Scientific, Waltham, MA).

## qRT–PCR

## All complementary DNAs (cDNAs) were generated as described in our previous reports. Briefly, 1 μg of total RNA, either fecal or cellular total RNA, was reverse-transcribed for single-stranded cDNAs using an oligo(dT)12–18 primer with the PowerScript® Reverse Transcriptase kit (Applied Biosystems), according to manufacturer protocol. Synthesized cDNA could be used directly in the following quantitative PCR analyses. In fecal and cellular samples, the levels of *RPS27L* (NM015920) mRNA were quantified relative to those of their own 18s ribosomal RNA (rRNA) (X03205) with TaqMan Master Mix (Roche Diagnostics), according to manufacturer instructions. The specific TaqMan probe for *RPS27L*, Hs00955038_g1, was purchased from Applied Biosystems Inc. Relative expression levels to 18s rRNA (primers for qRT-PCR: 5’-CTCAACACGGGAAACCTCAC-3’ and 5’- CGCTCCACCAACTAAGAACG-3’; universal probe number, #77) of the three DNA repair-related genes *E2F1* (NM005225, primers for qRT-PCR: 5’-TCCAAGAACCACATCCAGTG-3’ and 5’-CTGGGTCAACCCCTCAAG-3’; universal probe number, #05), a positive regulator of S-Phase entry, and *RAD51* (NM002875, primers for qRT-PCR: 5’-ATCACTAATCAGGTGGTAGCTCAA-3’ and 5’-CCCCTCTTCCTTTCCTCAGA-3’; universal probe number, #58) and *PRKDC* (NM006904, primers for qRT-PCR: 5’-TGCCAATCCAGCAGTCATTA-3’ and 5’-CGTGCCACAGCCACATAGT-3’; universal probe number, #08), which are related to VP16-induced double-strand break (DSB), were also measured using probes from the Universal Probe Library and TaqMan Master Mix. The PCR kinetics to calculate the quantitative data were analyzed using the LightCycler Software (version 4.05, Roche Diagnostics). Each run of fecal analyses also included an appropriate and predetermined diluted cDNA from a colonic cell line as a standard, to estimate the relative expression levels.

## Immunodetection of p53 and RPS27L

For immunohistochemistry, 4 μm sections from six paraffin-embedded colonic specimens were each stained with antibodies directed against p53 and RPS27L. Two independent pathologists reviewed the imaging results and confirmed the diagnosis. To analyze cellular RPS27L expression, cultured LoVo cells in six-well dishes for immuncytochemisrty were fixed, permeabilized, and blocked. Briefly, cultured cells were incubated for 1 h with an anti-RPS27L antibody (diluted 1:2000) at 37 °C and then for 30 minutes with a biotinylated anti-rabbit antibody (1:600) at room temperature. The staining of RPS27L in the cells was visualized with the Vectastain ABC kit and a diaminobenzidine tetrahydrochloride solution (both from Vector Laboratories, Burlingame, CA), according to the manufacturer’s instructions. The cells were counterstained with hematoxylin and mounted with mounting medium (Vector Laboratories). To identify the changes in the cellular distribution of RPS27L after treatment with the antitumor drug VP16 (etoposide), nuclear extracts from colonic cells were harvested using the NE-PER Nuclear and Cytoplasmic Extraction Reagent Kit (Thermo Fisher Scientific), according to the manufacturer’s instructions. In addition, the level of each protein was determined with immunoblotting. The cell lysate of each cell line was harvested by scraping off the cells in a radioimmunoprecipitation assay (RIPA) buffer (50 mM Tris-HCl, pH 7.4; 1 mM EDTA; 150 mM NaCl; 1% Nonidet P-40; 0.5% sodium deoxycholate; and proteinase inhibitors). Immuno-blots of each protein were performed using a standard procedure. Briefly, 10 μg of protein from each sample was mixed with a reducing NuPAGE sodium dodecyl sulfate (SDS) sample buffer (Life Technologies), denatured for 5 min at 95°C, separated by 12–15% SDS–PAGE, and blotted onto a polyvinylidene difluoride membrane (Amersham Biosciences). With the exception of an anti-RPS27L antibody, which was produced as described in our previous report, and the antibody for anti-lamin A/C (sc-7292, as a nuclear control) was purchased from Santa Cruz Biotechnology (Santa Cruz Biotechnology, USA) in distinguishing RPS27L in different compartments of VP16-treated LoVo cells, as indicated. To strengthen RPS27L relevance in apoptosis, antibodies for anti-PARP (#9546, Cell Signaling) and anti-GAPDH (AM4300, Ambion) were used after exposure of different LoVo cells with VP16 (10 μM or 50 μM) for 48 h. Various titers were used to determine the expressions of RPS27L (1:1000), lamin A/C (1:1000), PARP (1: 1000), and GAPDH (1: 4000). Most Western blots, except for the detection of cleaved PARP, were directly developed using the Western Blot Chemiluminescence Reagent (PerkinElmer Life and Analytical Sciences), according to manufacturer instructions. To detect the cleaved PARP sensitively, the immuno-blot was first stained with Ultra-Sensitive ABC Peroxidase Staining Kits (Thermo Scientific), following manufacturer directions before chemiluminescent development. All images were acquired using the [FluorChem FC2](http://www.bucher.ch/en/products/cellbiosciences/Western_Blot_Imaging/FluorChem_FC2.html) system (Cell Biosciences, Santa Clara, USA).

## Flow Cytometry

Propidium iodide (PI)-stained DNA and bromodeoxyuridine (BrdU) incorporation were used to determine the cellular DNA content and active DNA synthesis, respectively. To synchronize the cells, 1.25 × 10^5^ stable colonic cells in each well of six-well plates were starved in serum-free medium for 24 hours and then restimulated with fresh medium supplemented with 10% fetal bovine serum and 10 μM VP16 for 24 hours. Then 40 μM BrdU (Sigma) was pulse-incorporated into these VP16-treated cells for 1 hour and the cells were labeled with an indirect immunofluorescence staining kit (BD Biosciences, San Jose, CA), according to the manufacturer’s protocol, with a minor modification. Briefly, the fixed cells were incubated for 30 minutes in PBS containing 0.2 mg/mL RNase A, 0.1% Triton X-100, and 20 μg/mL PI at room temperature. Fluorescence emitted at 585 nm from the PI-stained DNA and at 530 nm from BrdU–fluorescein isothiocyanate (FITC)-positive DNA was detected with a FACScan flow cytometer (BD Biosciences). The percentages of cells (from a total of 10^4^ cells) in the different cell phases and the percentage of BrdU-positive cells were determined using FlowJo 8.7 software.
